# Neuromuscular Junction as an Entity of Nerve-Muscle Communication

**DOI:** 10.3390/cells8080906

**Published:** 2019-08-16

**Authors:** Elisa Lepore, Irene Casola, Gabriella Dobrowolny, Antonio Musarò

**Affiliations:** Laboratory affiliated to Istituto Pasteur Italia–Fondazione Cenci Bolognetti, DAHFMO-Unit of Histology and Medical Embryology, Sapienza University of Rome, Via A. Scarpa, 14, 00161 Rome, Italy

**Keywords:** NMJ, muscle-nerve interaction, ALS, aging, PKC

## Abstract

One of the crucial systems severely affected in several neuromuscular diseases is the loss of effective connection between muscle and nerve, leading to a pathological non-communication between the two tissues. The neuromuscular junction (NMJ) represents the critical region at the level of which muscle and nerve communicate. Defects in signal transmission between terminal nerve endings and muscle membrane is a common feature of several physio-pathologic conditions including aging and Amyotrophic Lateral Sclerosis (ALS). Nevertheless, controversy exists on whether pathological events beginning at the NMJ precede or follow loss of motor units. In this review, the role of NMJ in the physio-pathologic interplay between muscle and nerve is discussed.

## 1. Introduction

### 1.1. Muscle and Nerve Communication: A Peer to Peer Dialogue at the Neuromuscular Junction

Muscle and nerve communicate at the level of a specialized region, namely the neuromuscular junction (NMJ), a synaptic connection where the peripheral nervous system contacts skeletal muscle fibers, governing crucial vital processes, such as body voluntary movements and breathing [1]. Nerve activity guarantees not only muscle contraction but can induce myoblast orientation [2] and strictly influences fiber type specification and myosin isoforms expression [3]. Skeletal muscle fibers can be generally classified as fast or slow twitch, based on their contractile and metabolic properties [4]. These properties are dependent on the pattern of motor nerve stimulation. Tonic motor neuron activity promotes the slow fiber phenotype, while infrequent motor neuron firing results in fast fibers generation [5]. Cross reinnervation experiments demonstrated that fast muscles turn into slow ones when reinnervated by a slow nerve, whereas slow muscles turn into fast ones when reinnervated by a fast nerve [6,7]. Of note, skeletal muscle is also a source of signals that influence neuron survival, axonal growth, and maintenance of synaptic connections. Indeed, the development in the absence of skeletal muscle results in the sequential ablation of motor neurons in the spinal cord and brain [8]. In addition, it has been demonstrated that during the stage of reinnervation the inducible depletion of adult satellite cells (SCs), the classical muscle stem cell compartment, can impair NMJ regeneration and affect NMJ morphology [9]. This suggests that skeletal muscle and nerve influence each other in a functional dialogue that is fundamental for their survival and mechanism of action. The loss of effective connection between muscle and nerve leads to a pathological non-communication between the two tissues. In this context, NMJ is the central player of the physiopathologic interplay between muscle and nerve.

### 1.2. Perisynaptic Schwann Cells, a Third Speaker in the Dialogue

NMJ is composed by three major elements: the presynaptic region containing the nerve terminal, the synaptic cleft, and the postsynaptic surface referred to as the endplate. Several studies have recently pointed out a critical cellular component in the neuromuscular synapse, namely the synapse-associated glial cells also called Perisynaptic Schwann Cells (PSCs).

PSCs play an active role in NMJ development and in the maintenance and remodeling of adult neuromuscular endplate. It has been demonstrated that during development, Schwann cells migrate in association with growing axons and are located close to NMJ just before muscle-nerve contacts [10]. Moreover, in vivo experiments demonstrated that PSCs increase their number before the period of synaptic growth and play a guidance role during axon growth and innervation. In contrast, lacks of PSCs can induce a complete loss of synapses during development or determine pre- and post-synaptic defects in developing NMJ [11,12].

In adult organisms, PSCs ensure NMJ stability, changing their properties according to the state of innervation [13]. PSCs provide several trophic molecules, such as agrin, transforming growth factor beta 1 (Tgf-β1), and Matrix Metalloproteinases (MMPs) that can support muscle-nerve contact and promote AChR clustering [10,14]. In turn, PSCs are influenced by muscle derived factors, such as NT-3, Neuregulin-1, or by neuronal released factors, such as ATP, Ach, and neuronal agrin [14].

### 1.3. Kranocytes Cells, Capping Cells at the NMJ

An additional junctional cell type element, along with skeletal muscle fibers, motor neuron terminals, perisynaptic terminal Schwann cells, is a fibroblast-like cell, named kranocyte [15]. Kranocyte, lying outside the synaptic basal lamina, caps the NMJ above the perisynaptic Schwann cells and extends its cytoplasmic processes over the end-plate area [15,16]. In newborn mouse muscle, kranocytes are equally distributed, whereas during mouse postnatal development they become restricted at the endplate zone [15]. Whole mount immunostaining experiments demonstrated an unequivocal disposition of this subpopulation and elucidated that kranocytes are not Schwann cells. They express a specific immunocytochemical profile, including collagen-synthetizing enzyme and neuregulin [15]. In this context, kranocytes may interact with terminal Schwann cells through neuregulin mediated signaling [17].

Although in adulthood kranocytes remain confined at the NMJ region, they play a role in nerve repair and regeneration after denervation or paralysis. Within 24 h of denervation, kranocytes proliferate and spread throughout the NMJ area, even before the sprouting of Schwann cells [15,17], suggesting that this different time of reaction may promote and trigger terminal Schwann cells outgrowth and activation [15]. Thus, mammalian kranocytes capping cells are integral player of neuromuscular functionality.

## 2. ALS and Aging as Paradigmatic Examples of Altered Nerve-Muscle Communication

The impaired neuromuscular transmission represents a critical feature of several pathological conditions in which structural changes in NMJ might contribute to muscle weakness, altered motor neuron activity, and loss of muscle fibers.

In this review, we will analyse two extreme physio-pathologic conditions, namely aging and Amyotrophic Lateral Sclerosis (ALS).

Aging represents a physiologic and progressive decay of the homeostatic processes of the entire organism and it is broadly defined as the time-dependent functional decline that affects most living organisms. Common denominators of aging in different organisms include genomic instability, telomere attrition, epigenetic alterations, loss of proteostasis, deregulated nutrient sensing, mitochondrial dysfunction, cellular senescence, stem cell exhaustion, and altered intercellular communication [18].

ALS is a complex and severe disease associated with numerous pathologic mechanisms, including oxidative stress, mitochondrial dysfunction, axonal damage, microglial activation, inflammation, excitotoxicity, and protein aggregation [19].

**Figure 1:**
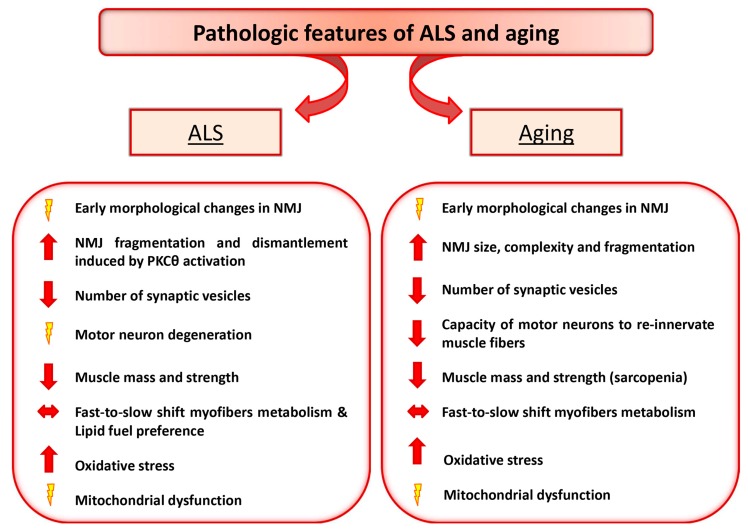
Amyotrophic Lateral Sclerosis (ALS) and Aging share some common pathologic features. The diagram depicts the relevant pathologic changes observed in ALS and aging.

Interestingly, although aging and ALS display different progressive loss of physiological integrity, they share some common pathologic feature, including high levels of oxidative damage, decreased number of synaptic vesicles, reduced and altered mitochondria in the plaque region [20,21,22,23,24] (Figure 1). Moreover, an early pathologic sign observed in both ALS and aging is the morphologic alteration of NMJ. However, whether changes in the NMJ precede or follow loss of motor units remains unresolved. Here we detail the NMJ degenerative features in ALS and aging and discuss the paradigm that retrograde signaling, from muscle to nerve, might represent an early event preceding motor neuron degeneration.

### 2.1. Motor Neuron Diseases: ALS

One of the best examples of impaired interplay between nerve and muscle is ALS, a fatal disease characterized by motor neurons degeneration, muscle atrophy, weakness and, ultimately, muscle paralysis with respiratory failure. During ALS progression muscle denervation is accompanied by changes in muscle fiber profile with a preference loss of fast-twitch fiber [25,26], due to higher vulnerability of fast-fatigable innervating motor neurons and to specific signals released by PSCs [27]. ALS is epidemiologically classified into two forms: sporadic (90–95%) and familial (5–10%) form. Among the familial cases, approximately 20% correlate with mutations in the sequence of the Superoxide Dismutase 1 gene (*SOD1*), which encodes for an important antioxidant enzyme. In addition to *SOD1* mutations, other gene defects have been reported to cause ALS, including *senataxin* (*SETX*) [28], *alsin* [29,30], *dynactin* [31], *synaptobrevin/VAMP* (vesicle-associated membrane protein)-associated protein B (*VAPB*) [32], *TDP-43, FUS/TLS* [33,34], *profilin* (*PFN1*) [35], *MATR3*, *CHCHD10*, *TBK1*, *TUBA4A*, *NEK1*, *C21orf2*, and *CCNF* [36]. However, even in these cases, where a well-defined mutation has been linked to the disease, a clear correlation between the genetic defect and the pathophysiology of the disease has not yet been disclosed.

Much of what we presently know about the role of mutant genes in ALS is based on studies of transgenic animals in which the potential genes involved in ALS are overexpressed under the control of specific promoters. Nevertheless, the failure to translate the positive results obtained in animal models into successful trials in human has cooled the enthusiasms and raised important questions on the validity of either animal models or methodological approaches. Thus, robust criteria and guidelines for preclinical animal research in ALS are necessary [37]. One of the experimental models that has been widely used in ALS-related studies is the transgenic mutant SOD1 mouse. Although the SOD1 mutant mice present some limitations compared to ALS patients it remains, along with other ALS-related mice, an ideal model for preclinical tests and proof-of-concept studies [38]. The obvious loss of motor neurons in the spinal cord initially focused attention on how mutant SOD1 may act within motor neurons to provoke neuronal degeneration and death. Among pathogenic events, glutamate-induced excitotoxicity, oxidative stress, protein aggregation, and mitochondrial dysfunction within motor neurons have been proposed. However, the mutant gene products are widely expressed, raising the possibility that the toxic cascade may be achieved wholly or in part by mutant SOD1 action in non-neuronal cells. Notably, restriction of *SOD1* mutant expression selectively to post-natal motor neurons failed to produce detectable sign of pathology or motor-neuron disease [39], suggesting that other cell types may be involved in ALS-associated neurodegeneration. Indeed, analysis of chimeras generated between wild type and SOD1 mutant mouse embryonic cells revealed that wild type non neuronal cells in adult chimeric animals extended the survival of SOD1 mutant motor neurons [40].

The generation of mouse models expressing human mutated *SOD1* gene (*SOD1^G93A^*), exclusively in skeletal muscle, added new insights into the potential primary targets of the mutant SOD1 toxic protein. Muscle-specific expression of mutant *SOD1^G93A^* caused accumulation of reactive oxygen species (ROS), mitochondria dysfunction, muscle atrophy, NMJ dismantlement, microgliosis [23,41], and neuron degeneration [42]. All together this evidence suggests that local toxic effect of *SOD1^G93A^* is a primary determinant of ALS-associated muscle pathology and that retrograde signals from muscle to nerve may contribute, in a sort of dying back phenomenon, to synapse and axon damage [43,44]. In fact, several other recent evidences confirmed the hypothesis that motor neurons are not the only primary targets of SOD1^G93A^-mediated toxicity and revealed that early changes of neuromuscular transmission start long before motor symptoms onset. Fischer and colleagues demonstrated that pathological changes of motor neuron disease begin at the distal axon and they observed denervation and reinnervation changes in muscle tissue without any pathological signs in neurons cells [45]. Furthermore, Schafer’s group characterized two different types of motor neurons, the “losers” or denervated branches, and the “compensators”, or reinnervating branches, which display different “susceptibility” to the toxic properties of *SOD1^G93A^* mutant gene product [46]. Although a conclusive link is still missing, it is intriguing to speculate that loser and compensator neurons are subject to different influences from neighboring interneurons, astrocytes, or microglia [40] and from the vasculature [47,48] and muscle fibers they innervate, all of which might provide either toxic or protective factors.

This evidence supports the notion that NMJ dismantlement can occur independently from motor neuron degeneration and may represent an early pathogenic signature of muscle-nerve communication defects.

#### 2.1.1. PKCθ as a Potential Signaling Involved in NMJ Dysfunction in ALS Disease

It has been demonstrated that Protein Kinase C and A (PKC and PKA) activities have antagonistic effects on NMJ stability and Acetylcholine Receptors (AChRs) recycling [49]. Stimulation of PKC or inactivation of PKA significantly accelerates the removal of postsynaptic AChRs and depresses AChR recycling. A recent work disclosed the PKC isoform, namely PKCθ, which triggers NMJ dismantlement in a mouse model expressing *SOD1^G93A^* mutant gene. PKCθ is a kinase that triggers in physiological condition the dismantlement of supernumerary NMJs during the first postnatal days [23,50]. It has been demonstrated that perturbation in redox signaling cascades, induced by muscle-specific accumulation of mutant *SOD1^G93A^* gene, promotes the activation of PKCθ that in turn acts as mediator of endplates destabilization [23]. In contrast, PKCθ selective pharmacological inhibition reduces oxidative damage and guarantees the stabilization of AChR turnover and the rescue of the NMJ morphological complexity [23].

PKCθ also modulates the activation of two sensors of nerve activity, Calcineurin (CN) and Nuclear Factor of Activated T cells (NFATc1), which promote slow muscle phenotype through the activation of their target Myocyte Enhancer Factor 2D (MEF2D), involved in muscle glucose homeostasis. Furthermore, it has been demonstrated that PKCθ-lacking mice display an impaired glucose homeostasis in fast muscles [51,52], suggesting that PKCθ can participate to muscle metabolic changes associated to muscle denervation and ALS disease progression. 

#### 2.1.2. Metabolic Changes and Mitochondrial Alteration May Influence NMJ Stability

Metabolic homeostasis and energy balance result severely compromised in both ALS patients and mouse models, where the energy expenditure is higher than the intake and it is associated to an increased energy demand and to an abnormal lipid metabolism [53,54,55,56,57]. In fact, a recent study revealed that ALS patients start losing weight almost 10 years before the clinical onset of the disease and show a higher daily energy intake to compensate the elevated energy consumption [58]. Likewise, the ALS mouse model that ubiquitously overexpress *SOD1^G93A^* mutant gene shows similar energetic alterations, such as an increased energy expenditure and a concomitant skeletal muscle hypermetabolism [59].

Further evidence has also pointed out that metabolic alterations in SOD1^G93A^ mouse model can be distinct from denervation [60] and that muscle specific accumulation of SOD1^G93A^ is able to induce metabolic changes in glucose and lipid pathways, independently from motor neuron degeneration and preceding muscle denervation [61]. These data suggest that some of the metabolic changes represent part of the earliest signs of morphological and functional alterations occurring in the motor nerve terminals. Among cell components, dysfunctional mitochondria represent key regulators of metabolic changes [62,63,64,65]. Indeed, treatment with mitochondria protective drug can preserve NMJ function and structure in ALS mouse model at late stages of the disease, suggesting a central role of mitochondria in the pathology [66]. The link between muscle functional mitochondrial alterations and neurodegeneration has been clarified by Dupuis and collaborators. The analysis of a transgenic mouse model overexpressing the potent mitochondrial uncoupler protein (*UCP1*) exclusively in skeletal muscle revealed that muscle specific expression of *UCP1* induces an age-dependent deterioration of the NMJ [67]. This defect correlated with progressive signs of denervation, supporting the idea of the crucial role of skeletal muscle on nerve homeostasis and revealing the potential molecular signature associated with the dying back phenomenon [67].

It has been demonstrated that antioxidant treatment of mice expressing human mutated *SOD1* gene (*SOD1^G93A^*) exclusively in skeletal muscle was able to rescue mitochondrial functionality and NMJ stability [23,41]. Moreover, studies on SOD1^G93A^ mice overexpressing the neurotrophic factors GDNF or IGF-1 in muscle tissue revealed an hyperinnervation of muscle fibers [68,69,70,71,72,73,74,75,76], NMJ stability, and motor neuron preservation [40,44,47]. This suggests that a sort of “saving back” approach can be envisaged. Nevertheless, not all treatments guarantee the same efficacy. Neuromuscular Magnetic Stimulation (NMMS) was able to induce a significant increase of muscle strength, without significant change in Compound Muscle Action Potential (CMAP) amplitude, suggesting that the improvement was not related to reinnervation phenomena [77]. In particular, NMMS activated a molecular mini-circuit to counteract muscle atrophy, to promote muscle robustness and maintenance of fiber type composition, and to induce NMJ stabilization. In addition, NMMS induced an improvement of AChR functionality, characterized by the reduction of the expression level of gamma subunit of AChR [77], whose expression increases in denervated muscle or under conditions that alter NMJ functionality [23,78].

It has been also demonstrated that Musk activation preserves synapses at the NMJ in diaphragm muscles of mutant SOD1 transgenic mice, without any positive effects on motor neuron death and survival [79]. Cleveland and colleagues demonstrated that the over expression of Peroxisome proliferator-activated receptor-gamma coactivator (PGC)-1alpha in the skeletal muscle of ALS transgenic mice is able to increase mitochondria energy production, ameliorating muscle endurance and performance and reducing muscle atrophy, without any effects on muscle innervation and motor neuron survival [80]. Nevertheless, the authors did not detail NMJ morphology in PGC1α overexpressing ALS mice even if it has been demonstrated that muscle-specific *PGC-1α* expression induces functional improvement in NMJ [81]. These data suggest that additional work is necessary to define the best experimental conditions to support a “saving back” therapeutic approach. Moreover, all these data reinforce the evidence that ALS is a complex multi-systemic disease in which alterations in structural, physiological and metabolic parameters in different cell types, including motor neurons, glia, NMJ, vasculature, and muscle, act synergistically to exacerbate the disease. Moreover, all these works suggest that a single treatment or the modulation of a single pathway may be not enough to significantly counteract motor neuron degeneration and increase survival. Combinatorial approaches on different tissue targets might be necessary to achieve satisfactory therapeutic benefits.

### 2.2. Aging and NMJ Defect

Another physio-pathologic condition where the functional communication between muscle and nerve is compromised is aging. As mammals grow older, many functional and structural changes occur in skeletal muscle and NMJ, leading to gradual loss of mobility [82].

It is widely accepted that not all muscles share the same susceptibility to age-related alterations. Several studies indicate diversity in muscle fibers susceptibility to the denervation process, based on motor neuron type. The NMJ associated with faster-contracting motor neurons are more susceptible to age-related structural changes than those associated with slower-contracting motor neurons [83,84,85].

One of the first evidences of the aging process occurring in NMJ is the formation of new branches of terminal axons that form new synaptic sites on muscle fibers [82]. NMJ undergo remodeling processes with cyclical extension and retraction of motor nerve terminals, leading to an increase in the complexity of the nerve terminal arborization [82]. In particular, this remodeling process is characterized by the increase in size and complexity of the branches of terminal axons [86], associated with fragmentation [87,88,89,90,91,92,93,94,95] and folds reduction of postsynaptic sites [90,96]. It has been demonstrated that oxidative stress, along with compromised mitochondria and increased intracellular calcium, amplifies the presynaptic decline in NMJ, accompanied with a decreased number of synaptic vesicles, similarly to what observed in ALS mouse models [23,91,97]. This initial NMJ dysfunction is followed by a neurodegeneration promoted by increased production of inflammatory cytokines and loss of trophic support [97]. Moreover, the evidence that some denervated fibers are not successfully re-innervated during aging raise the prospect that this alteration is at the base of the progressive decline in muscle mass and strength with aging, a condition known as sarcopenia.

The triggers of the NMJ alterations during aging are still debated. It is still unclear whether NMJ changes in aged muscles are caused by alterations in motor neurons or rather in skeletal muscle fiber [98]. One of the possible causes is the death of a fraction of motor neurons, occurring in humans between the age of 60 and 90 [99]. It has been suggested that motor neuron death provokes a temporary denervation of downstream muscles and induces a collateral reinnervation process. This process is driven by Perisynaptic Schwann cells that grow from the axon stump to guide surviving motor neurons that sprout their axons to create new synaptic terminals on muscle fibers [100,101]. Nevertheless, in advanced age, motor neurons show impaired capacity to re-innervated denervated fibers, suggesting that alterations in different components of nerve-muscle circuit impinge the potential compensatory mechanisms.

Other studies suggest that changes in the end-plate morphology and NMJ remodeling that occur with aging precede the loss of fast motor units and suggest the involvement of retrograde signaling. It has been reported that proteolytic cleavage of agrin, a proteoglycan involved in NMJ development, maturation, and AChRs clustering [102], induces early onset sarcopenia in young adult mice [103], whereas the injection of a neurotrypsin-resistant agrin fragment stabilized NMJ and improved the phenotype of neurotrypsin-overexpressing mice [104].

Other studies employed the effect of Muscle Electrical Stimulation (ES) to improve muscle functionality and to counteract NMJ decline during aging [105]. ES treatment in old sedentary people improved muscle performance, increasing fiber size, stimulating satellite cells and modulating the degeneration of the mitochondrial apparatus [106,107]. Moreover, it has been reported that ES treatment increases the number of fast twitch fibers [108] and counteracts neuromuscular disabilities from age related NMJ degeneration in paraplegic patients [109,110]. At molecular level ES treatment activates signaling pathways that decode for a specific calcium signaling involved in metabolic and structural adaptation of muscle fibers, such as the Calcineurin-NFAT and CamKII pathways that control the maintenance or switching of muscle fiber type [108]. Moreover, it has been described that biphasic electrical stimulation significantly increases the number and size of AChRs clusters available for NMJ formation during innervation [105] and that electrical stimulation promotes axonal growth and sensorimotor functional recovery after injury [106].

#### Factors that Influence NMJ Stability during Aging

Among the factors that can affect the stability of the NMJ there are metabolic changes associated to mitochondrial dysfunction and lifestyle.

Several recent evidence demonstrated that mitochondrial energy metabolism is altered during muscle aging, which is also accompanied by a reduction in the rate of oxygen consumption [107,111]. In human skeletal muscle, as well as in animal models, the mitochondrial capability to produce ATP decreases with age [112,113,114,115,116,117,118].

It is widely recognized that mitochondrial dysfunctions have a causal link with cellular ROS accumulation and therefore, as organisms grow old, there is an increase of intracellular ROS and consequently an accumulation of damaged cell components in all the tissues [119]. Since muscle and nerve are highly metabolic active tissues, their mutual communication is affected by mitochondrial dysfunction.

Another factor playing a crucial role in sarcopenia progression is lifestyle. Physical inactivity and impaired nutrition stimulate loss of muscle mass and NMJ defect, exacerbating sarcopenia [120,121]. It has been demonstrated that exercise induces muscle hypertrophy and NMJ remodeling, and improves recovery of peripheral nervous terminal after nerve injury [122,123]. In old mice exercise training is able to minimize NMJ expansion that turn back to levels comparable to young mice [124].

How skeletal muscle and NMJ send retrograde signals to motor neurons represents an intriguing field of research. Skeletal muscle has been identified as an endocrine organ that produce and secrete growth factors, cytokines, and peptides, collectively indicated as myokines, which make muscle capable to communicate with other tissues and organs, including bone, intestine, adipose tissue, liver, pancreas, and brain [125]. One of the critical players that integrates muscle fiber function with motor neuron signaling following exercise is Peroxisome proliferator-activated receptor-gamma coactivator (PGC)-1alpha. PGC-1α expression activates a broad NMJ gene program and improves postsynaptic NMJ architecture [81,126]. It has been demonstrated that loss of skeletal muscle PGC-1α hampers acetylcholine receptor (AChR) clustering and the transcription of NMJ genes including AChRs, muscle-specific kinase, and utrophin [126].

Among other factors, Insulin-like Growth Factor-1 (IGF-1) plays a pivotal role in muscle growth, differentiation, and regeneration [127,128]. In mammalian organisms the majority of IGF-1 is produced and released by the liver as systemic growth factor, while a minor fraction is produced by other tissues and participates to autocrine and paracrine signals. In skeletal muscle, the binding of IGF-1 with its receptor activates anabolic, anticatabolic, and antiapoptotic signaling pathways that preserves muscle mass and strength [129]. It has been demonstrated that the overexpression of IGF-1 isoforms selectively in skeletal muscle maintains muscle mass and activates several pathways that promote the clearance of dysfunctional mitochondria and the maintenance of NMJ stability in senescence mice [130]. Moreover, it has been demonstrated that IGF-1 overexpression is sufficient to downregulate the levels of two cytokines, namely IL-1β and IL-6, in aged mice, counteracting the chronic inflammation process typical of aging (inflammaging) [130]. In this context, a crucial role is played by senescent Schwann cells that overexpress IL-6 cytokine and negatively affect the nerve microenvironment during muscle innervation [131].

## 3. Conclusions

**Figure 2:**
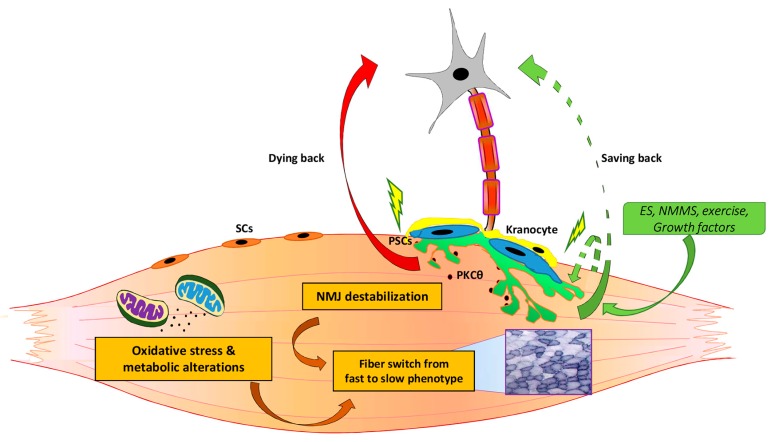
A summary of the relevant changes, cells, and signaling involved in the pathogenesis of ALS and aging. Morphological and functional alterations of the neuromuscular junction (NMJ) represent part of the earliest signs of aging and ALS. NMJ development, remodelling, and stability is also influenced by different signals and cells, including PSCs and kranocytes. According to the dying back hypothesis, and based on studies regarding how skeletal muscle interventions can influence NMJ, further studies should be encouraged to verify whether combinatorial approaches on different cell and tissue targets induce more satisfactory therapeutic benefits and to define whether a sort of “saving back” approach can be promoted to preserve NMJ integrity, to delay motor neuron impairment, and to counteract muscle-nerve dysfunction.

Significant advances have been provided in the understanding the critical components involved in aging and ALS. Morphological and functional alterations of the NMJ represent part of the earliest signs of aging and ALS. Nevertheless, no consensus has emerged to the cells, tissues, and pathways that are directly affected. Although several pieces of the aging and ALS pathogenic puzzles have been defined, the picture of these diseases remained to be completed. The field will benefit when the pathologic events, the factors, the cell and tissue components, and the late “restriction point”, which might trigger the final catastrophic event, will be sequentially characterized. This will also help to: (i) verify whether a sort of “saving back” approach can be envisaged and tested and to (ii) design more effective therapeutic approaches, acting in a timely fashion on the different cells and tissues involved in the diseases (Figure 2).
